# A Review on Electrospun Poly(amino acid) Nanofibers and Their Applications of Hemostasis and Wound Healing

**DOI:** 10.3390/biom12060794

**Published:** 2022-06-07

**Authors:** Yuexin Ji, Wenliang Song, Lin Xu, Deng-Guang Yu, Sim Wan Annie Bligh

**Affiliations:** 1 School of Materials Science and Engineering, University of Shanghai for Science and Technology, Shanghai 200093, China; 213353172@st.usst.edu.cn (Y.J.); wenliang@usst.edu.cn (W.S.); 212203153@st.usst.edu.cn (L.X.); 2 Shanghai Engineering Technology Research Center for High-Performance Medical Device Materials, Shanghai 200093, China; 3 School of Health Sciences, Caritas Institute of Higher Education, Hong Kong 999077, China

**Keywords:** amino acids, hemostasis, electrospinning, wound dressing

## Abstract

The timely and effective control and repair of wound bleeding is a key research issue all over the world. From traditional compression hemostasis to a variety of new hemostatic methods, people have a more comprehensive understanding of the hemostatic mechanism and the structure and function of different types of wound dressings. Electrospun nanofibers stand out with nano size, high specific surface area, higher porosity, and a variety of complex structures. They are high-quality materials that can effectively promote wound hemostasis and wound healing because they can imitate the structural characteristics of the skin extracellular matrix (ECM) and support cell adhesion and angiogenesis. At the same time, combined with amino acid polymers with good biocompatibility not only has high compatibility with the human body but can also be combined with a variety of drugs to further improve the effect of wound hemostatic dressing. This paper summarizes the application of different amino acid electrospun wound dressings, analyzes the characteristics of different materials in preparation and application, and looks forward to the development of directions of poly(amino acid) electrospun dressings in hemostasis.

## 1. Introduction

**Figure 1:**
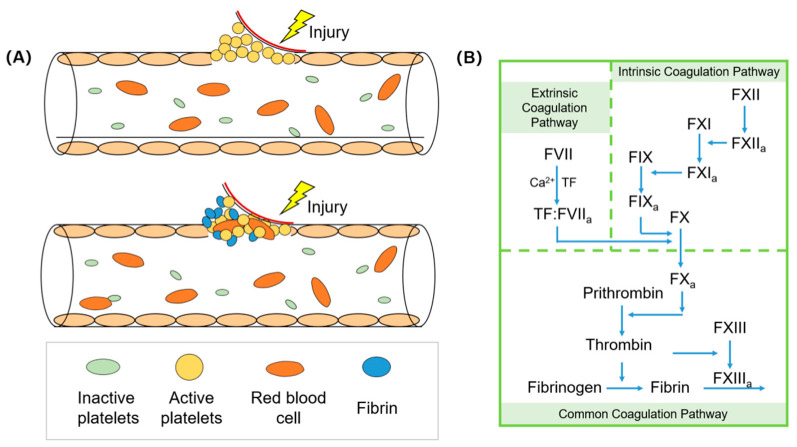
Mechanism diagram of coagulation process. (**A**) Hemostatic simulation diagram of damaged vascular model; (**B**) an overview of the coagulation cascade.

Coagulation is a complex process from unstable platelet embolism to stable insoluble fibrin in plasma. The coagulation process requires a combined and integrated response from all parts of the human body. In the initial hemostatic stage, platelets will form an initial platelet plug [1,2]. Platelets interact with von Willebrand factor (VWF), first binding to collagen, and then stably adhering to damaged vascular endothelial cells. When platelets are activated, they trigger the aggregation of other locally activated platelets. Platelets gather through the fibrinogen bridge, produce fibrin clots through the action of thrombin, and finally contract to form a tightly packed thrombus [3,4], as shown in Figure 1. Thrombin plays a central role in activating coagulation factors and platelets. When the secondary hemostatic stage is reached, the coagulation cascade includes intrinsic and extrinsic coagulation pathways, which are initiated by different substances and different substances and factors and are two different response pathways.

The coagulation pathways of the human body mainly include intrinsic hemostasis and extrinsic hemostasis. Intrinsic coagulation refers to that all the coagulation factors involved come from the blood, which is usually activated due to the contact between the blood and negatively charged foreign bodies. When blood comes into contact with a foreign body surface, the coagulation factor FXII first binds to the foreign body surface and is activated to FXIIa due to the negative charge of the foreign body surface. At the same time, the generated FXIIa can activate FXI to become FXIa, thus initiating the intrinsic coagulation pathway. In addition, FXIIa can promote the formation of FXIIa by activating prekallikrein. All coagulation factors involved in this clotting process come from the blood itself. On the contrary, when vascular trauma exposes tissue factor (TF) to the blood, the external pathway involving extrinsic coagulation factors begins [5,6].

Extrinsic coagulation refers to that not all the coagulation factors involved exist in the blood, and there are foreign coagulation factors involved in hemostasis. This process starts from the exposure of tissue factors to blood to the activation of FX. Due to the presence of calcium ions, TF is able to activate coagulation FVII. Then, the complex formed by the combination of TF and active FVII is able to continue activating FX, from activation of FX to the formation of fibrin clots. Then, the hydrolytic action possessed by thrombin is able to cleave fibrinogen, resulting in the formation of fibrin monomers [7]. The fibrin monomers are cross-linked with the involvement of Ca^2+^ and activated FXIII, eventually forming a solid fibrin clot that enhances platelet embolization during the initial hemostasis [8]. In the clinic, prothrombin time is used to reflect the status of the extrinsic coagulation pathway. The time required for extrinsic coagulation is short and the reaction is rapid. The extrinsic coagulation pathway is mainly regulated by tissue factor pathway inhibitor (TFPI). In addition, studies have shown that intrinsic and extrinsic coagulation pathways can activate each other [9].

Although materials that can directly activate the coagulation cascade can significantly improve hemostasis, they may cause systemic thrombosis. Metal ions, especially Ca^2+^, show a significant character in the coagulation process. Ca^2+^ is a cofactor that plays a universal role in the coagulation cascade. Important coagulation cascade steps such as the conversion of prothrombin to thrombin and the polymerization of monomers into fibrin involve Ca^2+^. Ca^2+^ is an imported cofactor in the coagulation cascade, accelerating platelet aggregation and activating the coagulation process, as well as enhancing material–wound adhesion. In addition, Ca^2+^ has a high water absorption rate and can be used to improve the plasticity of the material [10,11,12,13]. In addition, insufficient hemocompatibility can directly activate the coagulation process. Therefore, materials with passive access hemostatic mechanisms need to have surface properties such as hemocompatibility, antithrombotic, and anti-infective properties [14,15,16].

**Figure 2:**
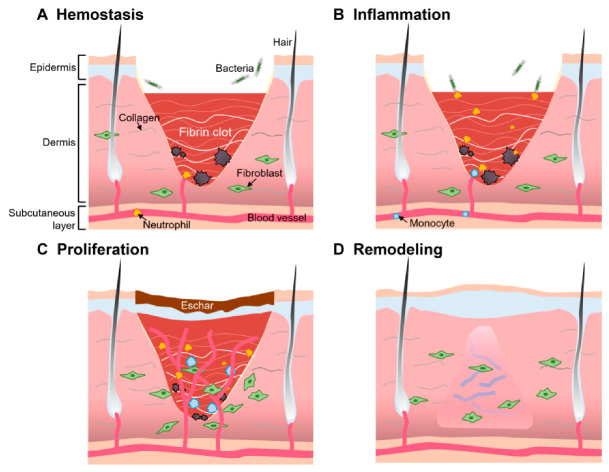
Four stages of wound healing. (**A**) Hemostasis; (**B**) inflammation; (**C**) proliferation; (**D**) remodeling.

The general theory of wound healing is divided into four stages, namely the hemostatic, inflammatory, proliferative, and remodeling stages of the wound. The barrier function of the skin is very important, serving to insulate, retain moisture, and protect the body from pathogens, so damage to the integrity of the skin can pose serious health risks to the body. In order to achieve rapid healing of damaged skin, the intrinsic healing process at the wound site begins immediately. Platelets will accumulate at the wound site and the fibrin clot will form at the same time, so blood flow will stop within a few minutes (Figure 2A). At this time, inflammatory cells such as neutrophils and monocytes are recruited to the wound site by locally released growth factors (GFs) and cellular mediators (Figure 2B). Removal of foreign bodies, bacteria, and damaged intrinsic tissues is the main task of the inflammatory response phase. When the phase is over, fibroblasts and epithelial cells are induced by macrophage GFs, proliferate, and migrate into the wound (Figure 2C). During the proliferative phase of the wound, new blood vessels gradually grow at the wound site, gradually producing enhanced collagen fiber, and granulation tissue consisting of epithelial cells, fibroblasts, and keratin-forming cells. However, complete wound healing is still a long-term process, taking weeks or months depending on the wound condition (Figure 2D). As the wound shrinks, granulation tissue is subsequently converted to a more stable ECM. Overall, wound healing time depends largely on the patient’s age, health status, and the presence of external factors such as the presence of an unremoved foreign body in the wound or the occurrence of a recurrent infection. Healing of acute wounds follows the above process and is completed within 8–12 weeks. However, for chronic wounds that take longer, they tend to stagnate in the inflammatory phase with massive exudation, severe infection, pain, and tissue necrosis, as in diabetic foot [17,18]. In such cases, chronic wounds often take longer to heal, even by years.

There are two views on wound healing, traditional clinical wound care believes that a dry environment with low humidity is conducive to wound healing and that oxygen in the air participates in the reproductive repair of wound tissue to accelerate the healing process. Therefore, the breathable dressing can achieve the purpose of more oxygen contact and make oxygen contact and make the cells proliferate rapidly. In fact, the dry healing method has obvious defects in theory: the cells in the wound do not react directly with oxygen in the air, which needs to be chemically combined with hemoglobin in the blood to be utilized [19,20]. Moreover, in actual clinical care practice, there are many disadvantages: the surface of the bed is dry and dehydrated, and the crawling of epithelial cells is hindered; the wound leaks rapidly, and the dressing needs to be changed frequently, so it cannot maintain a mild environment for the wound, which affects the process of cell reproduction and differentiation and makes the wound healing slow; the dry dressing is easy for the newly grown granulation tissue to stick to each other, and frequent changing is likely to cause secondary damage to the wound. The contact time between wound surface and air is long, and the dry healing dressing has large pores, which cannot effectively organize the invasion of bacteria and other harmful substances in the air and is prone to infection [21,22,23].

An ideal wound dressing usually needs to be non-antigenic, biocompatible, semi-permeable, biodegradable, flexible, and cost effective, and the wound dressing should preferably not adhere to the wound bed but should act on the surface of the wound. It must protect the wound from bacteria, infection, mechanical forces, and temperature, maintain a moist wound environment and be able to load certain drugs and active substances. Excellent wound dressings can effectively prevent wound infection and maintain the wound at the proper temperature and moderation in order to better promote surface-wound healing [24,25]. For severe burns, deep ulcers and other deeper wounds may not heal adequately due to the quality of the skin appendages not being good enough to produce a certain amount of regenerative buds. Dermal substitutes are an effective way to treat partially deep wounds, including decellularized and cell-seeded substitutes that can heal the defective site by promoting nearby cell migration. Full-layered wounds are a major problem in treatment because they contain not only epidermis and dermis but also subcutaneous fat and deep tissue, which can be more difficult to heal than superficial and partial thickness wounds. For these types of wounds, it is usually necessary to use autologous skin grafts or artificial skin substitutes for skin wound healing [26,27,28].

Electrospinning is able to structurally mimic the human ECM structure due to its nanoscale structure. At the same time, due to the high specific surface area, high porosity, and small size of nanofibers, they can be loaded with active ingredients that can promote therapy and can provide air exchange to the wound site and keep the healing environment moist. Electrospun nanofibers can also be personalized to the wound site, with handheld electrospinning enabling immediate clinical coverage of the wound [29], further enhancing the fit of the electrospun dressing to the wound [30,31]. The development of electrospinning technology in the field of hemostasis and wound healing has good prospects, and selecting amino acid-based polymers can largely enhance the biocompatibility of nanofibers for better utilization in biomedical and other fields [32,33].

**Figure 3:**
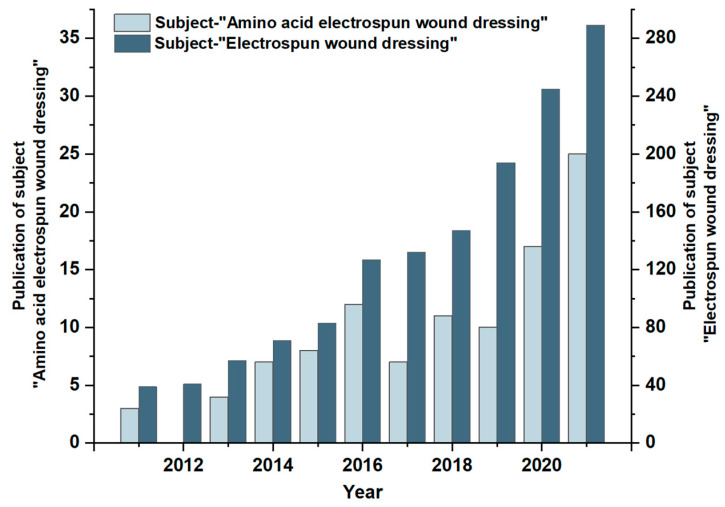
Statistics of literature retrieval on the “Web of Science” platform with the subject of “Electrospun wound dressing” and “Amino acid electrospun wound dressing”, respectively.

A search of the literature in recent years on “Electrospun wound dressing” and “Amino acid electrospun wound dressing” was carried out on the “Web of Science” platform. As shown in Figure 3, the number of publications on the topic of “Electrospun wound dressings” has been increasing year by year and has been maintained at more than 100 articles in recent years, indicating that the preparation of wound dressings by electrospinning has become a hot spot for research. The number of publications on the topic of “Amino acid electrospun wound dressings” is small but also on the rise, indicating that the development of amino acid materials in the field of electrospun wound dressings has certain prospects. This paper reviews the research progress of amino acid electrospinning materials in wound hemostasis and modification, and introduces the preparation technology of amino acid nanofibers and their latest applications in wound hemostasis and modification.

## 2. Electrospinning Technology

### 2.1. Introduction to Electrospinning Technology

Electrospinning differs from other spinning technologies, mainly in that the polymer solution or melt under the action of a high voltage electric field produces flow and deformation, generating a Taylor cone at the tip of the spinneret, and when the electric field strength is large enough to enable the droplets to overcome surface tension, a high-speed jet is generated, which is deposited on the receiving device to obtain fibers after a short distance electric field force of high speed stretching, solvent volatilization, and curing [34,35,36]. There are four main components in the electrospinning device, a high-voltage power supply that can generate up to 30 kV to higher voltages, a high-precision micro-pump with flow control, a nozzle, and an aluminum foil collector for fiber collection. The principle is that a polymer solution of a certain viscosity is loaded into a syringe with a metal nozzle, fixed to a metering pump, and a high-voltage electrostatic field is applied to the nozzle and the collector. When the voltage exceeds the critical value and the electrostatic force is greater than the surface tension of polymer droplets, a jet is generated at the spinneret, and the polymer solution is injected into the collector from the tip of the nozzle to form a nanofiber film. Fiber prepared by electrospinning is generally in the nanometer size and has extremely important applications in various fields due to its small size, large specific surface area, high porosity, large aspect ratio, and continuous uniformity stability.

Nanofibers have gained wide attention and applications in drug release, trauma recovery, and biological tissue engineering because of their small nanoscale size, large porosity, and high specific surface area. The diameter of nanofibers is smaller than that of cells, which can simulate the natural ECM in terms of structure and physiological function. It is also because of the nanoscale size of nanofibers that most tissues and organs in the human body are similar in form and structure to nanofibers, which also provides the possibility of biological tissue and organ repair [37,38]. At the same time, we also need to focus on the relationship between hemostatic materials and human body reactions. The use of materials with good biocompatibility allows better treatment without rejection, and materials with good biodegradability can reduce the pain of patients when removing dressings.

Electrospinning, as a technology that makes nanotechnology possible, allows not only the preparation of single polymer nanofibers but also the blending of multiple polymers and loading them with bioactive substances, and is widely used in wound healing. The extracellular matrix of the skin is composed of collagen, elastin, laminin, and various polysaccharides and proteoglycans together as fibrous structural proteins. Nanofibers with a composition and structure/system similar to that of ECM in skin tissue can be fabricated by electrospinning. Electrospun nanofibers can modulate skin cell proliferation, migration, differentiation, and extracellular matrix deposition responses [39,40,41]. Because of these unique properties, the nanofibers can be used as surgical sutures, wound dressings, and other fields, as well as wound healing and tissue engineering. Infection prevention is achieved by antimicrobial agents to nanofiber dressings or sutures since bacteria have developed some resistance to most antimicrobial agents such as antibiotics [42,43,44].

For synthetic or natural polymers with biodegradability electrospinning, nanofibers have been used to prepare nanofibrous membranes. These nanofibrous membranes have an extracellular matrix-like structure that provides a template for the proliferation of skin cells, thereby stimulating tissue regeneration. Electrospun nanofiber membranes have high porosity and large specific surface area, which can absorb the wound exudate in time, promote the transfer of nutrients in the wound environment, maintain the gas exchange at the wound site, and prevent the wound from dehydration [45,46]. In addition, the nanofiber membrane is soft, can be tailored to the size and shape of the wound, and is highly pliable; the nanofiber membrane protects the wound on a physical level and is able to protect the wound from other injuries and invasion by foreign microorganisms [47,48,49,50,51]. Electrospun nanofiber membranes can also be used as drug carriers to load anesthetics, antibacterial agents, bioactive molecules, etc., depending on different types of skin wounds (e.g., burns, trauma, chronic disease ulcers, etc.) [52,53]. It is made to function while controlling the release of drugs and promoting the wound healing process [54,55]. In addition, nanofiber film as a wound dressing needs to be in direct contact with the wound surface and can be sterilized by radiation or UV to ensure its sterility. By immobilizing blood cells, platelets, and other clotting factors, the nanofiber matrix, which has a structure similar to that of natural fibers, provides control of bleeding [56,57]. There are many ways to prepare nanofiber matrices, but the most traditional method is electrospinning.

### 2.2. Electrospinning Classification

**Figure 4:**
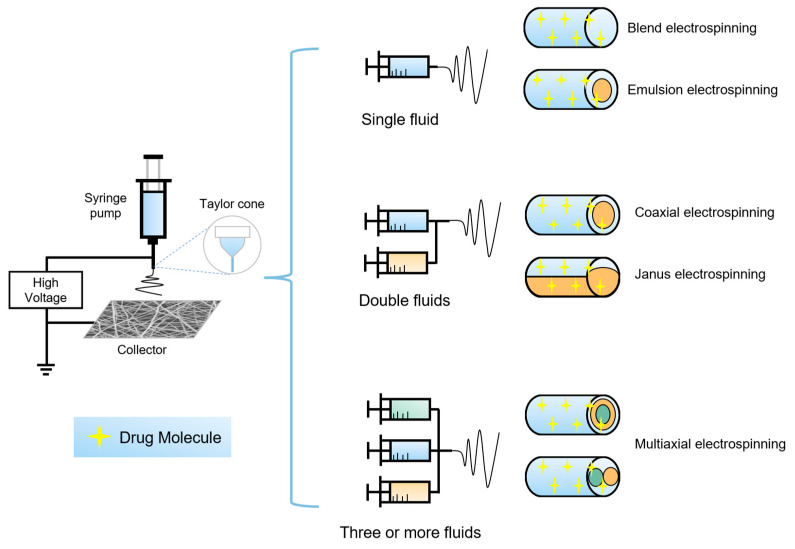
Electrospinning device and classification of electrospinning according to fluid quantity and spinneret structure.

Electrospinning can be classified into single fluid electrospinning, double fluid electrospinning, and multi-fluid electrospinning based on the type of spinning solution used in electrospinning. According to the geometry of the spinneret or nozzle that controls the production of nanofibers with different morphological structures, single-fluid electrospinning can be divided into blend electrospinning and emulsion electrospinning, and double-fluid electrospinning into coaxial electrospinning and Janus electrospinning and multi-fluid electrospinning. It is also possible to selectively load functional drugs during the preparation of spinning solutions, as shown in Figure 4.

#### 2.2.1. Single Fluid Electrospinning

Single-fluid electrospinning is the most basic production method of electrospinning, usually one or several polymers used are co-blended and dissolved in a solvent, and then inorganic nanoparticles, drugs, etc., that need to be added are added for electrospinning [58,59]. Single-fluid electrospinning is limited by the presence of only one spinning solution, thus also limiting the application of some good performance but non-spinnable polymer solutions [60]. Nasser et al. [61] loaded the antibiotic gentamicin sulfate and the local anesthetic lidocaine hydrochloride on PLLA nanofibers using poly(L-lactic acid) (PLLA) as the spinning solution substrate. In addition, the loading of aluminum chloride with a hemostatic effect on PLLA nanofibers can better utilize the hemostatic effect of nanofibers. Habiba et al. [62] co-blended chitosan, polyvinyl alcohol (PVA), and zeolite and performed electrospinning to obtain nanofibers with good adsorption and desorption rates, which can be well applied to wastewater treatment for the adsorption and purification of metal ions, etc. Single-fluid electrospinning is the most basic electrospinning method, on the basis of which two fluids and even multi-fluid electrospinning methods have also been developed, extending the application of electrospinning in various fields.

Emulsion electrospinning is a special single-fluid electrospinning method for obtaining nanofibers with a core-shell structure by only one solution. Electrospinning of emulsions produces nanofibers with a core-shell structure because the water and oil phases in the emulsion are delaminated, and when loaded with hydrophilic drugs, the drugs dissolved in the aqueous phase gradually diffuse into the emulsified oil phase and are contained in the core layer, eventually forming nanofibers with a core-shell structure. Parin et al. [63] firstly dispersed psyllium husk (PSH) in PVA and then blended it with D-limonene, which has a natural antibacterial effect, to prepare nanofibers by emulsion electrospinning. Hosseini et al. [64] used polyethylene glycol (PEG) as the aqueous phase and ethyl cellulose (EC) in ethyl acetate as the oil phase and encapsulated α-amylase in it to prepare nanofibers by the emulsion electrospinning method. This storage method of enzyme fixation has high stability, which can reach 20 times that of common free enzyme, and it is a novel and efficient enzyme immobilization system.

#### 2.2.2. Double Fluid Electrospinning

**Figure 5:**
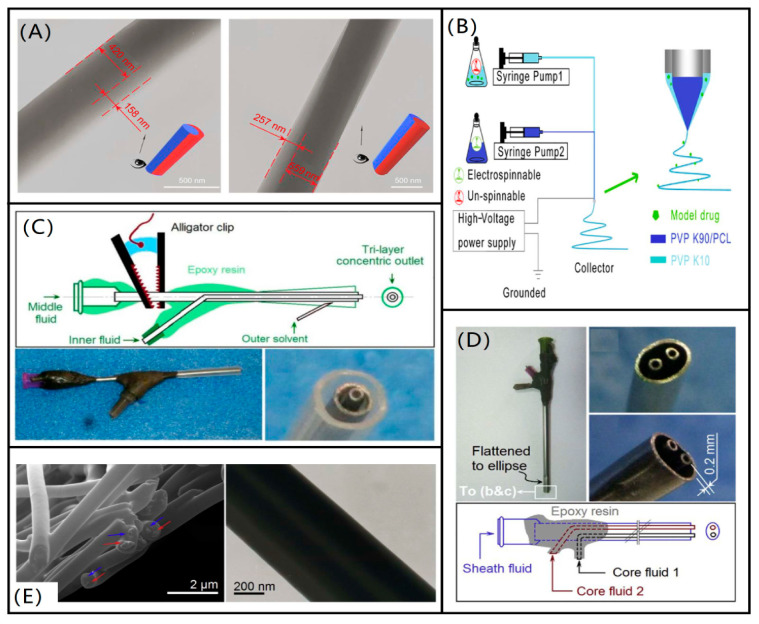
Nanofibers and spinnerets with different structures. (**A**) SEM pictures of nanofibers with Janus structure [66]; (**B**) production process diagram of coaxial electrospinning [73]; (**C**) schematic diagram of three-layer coaxial electrospinning spinneret [77]; (**D**) new three-fluid electrospinning spinneret [78]; (**E**) SEM images of nanofibers with sheath-separate-core nano-structure, as indicated by the red (The PEL-PAR core) and blue arrows (The ES-PAR core) [78].

The biggest difference between the Janus structure and the core-shell structure is that both sides of the spinning solution in the Janus structure make direct contact with air, and the two spinning solutions are in contact but with relatively little interfacial interaction, and this structure also suggests a new direction for the development and application of new nanomaterials. However, Janus fibers are not easy to prepare because the two parallel working solutions are introduced into the electric field with the same charge and usually repel and separate from each other. Therefore, the spinning solution and spinning conditions are carefully modulated. Yang et al. [65] prepared Janus wound dressings composed of polyvinylpyrrolidone (PVP) and ethyl cellulose (EC) polymers by parallel electrospinning, and the ciprofloxacin (CIP) and silver nanoparticles (AgNPs) were loaded on both sides. Firstly, the two-phase structure of the nanofibers could be clearly demonstrated by morphological observation, and then both in vitro dissolution experiments and antibacterial experiments could show that the added drug and metal nanoparticles could exhibit the advantages brought by the Janus structure in the prepared electrospun nanofibers. Zheng et al. [66] used ethyl cellulose (EC) and polyvinyl pyrrolidone K60 (PVP) as a two-phase solution and loaded with tamoxifen citrate (TAM) as the active drug component, and electrospinning was performed using eccentric spinneret. As shown in Figure 5A, a distinct Janus structure with one side round and one side crescent can be observed by scanning electron microscopy. Due to the presence of Janus nanostructure, it can release TAM rapidly in the first stage and slowly in the latter phase. This nanofiber with a Janus structure provides a new drug release system, which has a promising future in the field of controlled drug release and further applications.

Coaxial electrospinning technology was developed on the basis of electrospinning. The emergence of coaxial electrospinning technology can make up for certain polymers due to its electrospinning limitations, which cannot be stretched into fibers by ordinary electrospinning defects. Coaxial electrospinning has the advantage of further molding, can be easy to spin a polymer solution as the shell layer, cannot be spun or is difficult to spin a polymer solution as the core layer, in the electrospinning process, and the outer layer of the solution. In the electrospinning process, the outer layer solution acts as a template and the core layer solution is spun into fibers [67,68,69]. Coaxial electrospinning is mostly used in the medical field for the controlled release of drugs, many of which have poor water solubility and low dissolution rates. Combined with the small diameter, large porosity, and large specific surface area of nanofibers, it provides a rapid drug release method to promote the rapid dissolution of insoluble drugs [70,71]. To improve the viability of lactobacillus, Yu et al. [72] used polylactic acid (PLA) as the main raw material to produce nanofibers by coaxial electrospinning method, using PLA and fructooligosaccharides (FOS) as the shell solution, and lactobacillus plantarum was cultured in Man–Rogosa–Sharpe (MRS) agar to obtain the nucleation solution, which successfully encapsulated *Lactobacillus* into the nanofibers. This method successfully improved the viability of lactobacillus and proposed a novel encapsulation method for active substances. Li et al. [73] designed and prepared core-shell nanofibers with an ultrathin shell layer using the coaxial electrospinning technique, as shown in Figure 5B, with polyvinylpyrrolidone (PVP) K90 or polycaprolactone (PCL) as the core solution and selected drug and PVPK10 as the shell solution. The in vitro dissolution test confirmed that the core-shell nanofibers with ultrathin shells have good solubility and the drug in the fibers can be released within 1 min. This study can be used to prepare structured nanocomposites with ultrathin shells to enhance the rapid dissolution of insoluble drugs. Coaxial electrospinning has been intensively investigated in the fields of drug delivery and sustained release.

#### 2.2.3. Multi-Fluid Electrospinning

To adapt to more complex applications and to exhibit more diverse functions, multi-fluid electrospinning is gradually being widely studied. In the multi-fluid electrospinning process, at least one solution is required for electrospinning, which greatly enriches the variety and number of polymers involved in electrospinning and thus brings a new research boom [74,75]. One of the triaxial electrospinning processes is similar to coaxial but can further extend the capability of electrospinning in creating complex nanostructures. Yang et al. [76] used a spinnable ibuprofen and alcohol soluble protein mixture as the core solution, the nonspinnable cellulose acetate (CA) solution was used as the middle layer, and a nonspinnable acetone/acetic acid solvent as the outermost layer. The spinning solution flowing from the core layer of the spinneret is spinnable and can be electrospun to form nanofibers, while the CA in the middle layer is a non-spinnable solution that is deposited as a thin “nano-coat” on the core solution, while the external solvent has the characteristic of ensuring a stable and continuous spinning process. The presence of multi-fluids provides the possibility of more structural combinations for spinning, while the complex spinning conditions and spinning equipment require further in-depth study. Xu et al. [77] used a mixture of acetone, DMAc, and ethanol as solvent and dissolved different concentrations of metformin hydrochloride (MET) and CA as core and middle layer fluids, respectively, and the pure solvent was used as the outer layer fluid to prepare nanofibers by a modified three-layer electrospinning process, as shown in Figure 5C. This nanofiber was able to achieve a slow release of MET for drugs with water solubility and prolong the administration time of water-soluble drugs. Chang et al. [78] fabricated a novel tri-fluid spinning head with a shell enveloping two separate cores, as shown in Figure 5D. One shell solution consists of Eudragit^®^ E100 (EE) and paracetamol (PAR), and the two core solutions consist of Eudragit^®^ L100-55 (EL), PAR and Eudragit^®^ S100 (ES), PAR, respectively. Eudragit^®^ E100 (EE), Eudragit^®^ L100-55 (EL) and Eudragit^®^ S100 (ES) were purchased from Rohm GmbH & Co. KG (Darmstadt, Germany). This sheath-separate-core nano-structure combines the three spinning solutions, as shown in Figure 5E, and the characteristic structure can be observed by SEM images. This new electrospinning structure is able to release drugs according to different pH and is an intelligent responsive three-phase drug release system, which has good prospects for application in the field of controlled drug release. Along this way, some brand-new electrospun nanostructures can be further developed for biomedical applications [79].

### 2.3. Electrospinning Influence Factors

The factors affecting electrospinning are mainly divided into the nature of the spinning solution itself, spinning parameters, and environmental parameters. The nature of the spinning solution itself includes the molecular weight of the polymer, molecular structure, the nature of the solvent, the viscosity of the spinning solution, electrical conductivity, etc. [80,81,82], as shown in Table 1. The molecular weight of the polymer largely affects the viscosity and rheological properties of the polymer, which is intuitively reflected in the diameter of the nanofibers; generally speaking, the higher the molecular weight of the polymer, the larger the diameter of the nanofibers. A faster solvent evaporation rate will cause the fibers to not completely split and refine, and the fiber diameter will increase; while a slow solvent evaporation rate will also make the fibers stick to each other on the collection plate, and no complete nanofibers will be obtained; the faster the solvent evaporation rate, the larger the fiber porosity and specific surface area; the slower the solvent evaporation rate, the less easy to remove the solvent residue. In a certain range, the higher the viscosity of the spinning solution, the more likely a bead structure will be produced and block the spinneret; the lower the viscosity, the smaller the fiber diameter, but too low will produce an electrospray. The dielectric constant of the spinning solution will also have an impact on the nanofibers; in general, the higher the dielectric constant the smaller the diameter of the nanofibers, while a dielectric constant that is too small is also likely to produce bead fibers, which is not conducive to the preparation of nanofibers [83,84,85].

Spinning parameters include spinning voltage, liquid feed rate, collection distance, etc. [86]. The higher the spinning voltage, the higher the tensile strain rate, and the smaller the nanofiber size will be; too much voltage will increase the speed of the fiber ejection so that the solvent cannot be fully volatilized, which will also lead to spinning instability; too little voltage will cause the appearance of solution droplets, and the fiber diameter will be coarse [87]. For the feed rate, generally speaking, the greater the flow rate the larger the fiber diameter; a feed rate that is too low will easily interrupt the spinning process; and a feed rate that is too large will produce an unstable Taylor cone, and the droplets will directly drop down. The distance from spinneret to collector also affects nanofibers, the fiber cannot be fully stretched, and the solvent cannot be completely evaporated; a distance that is too large will affect the electric field strength and will also affect the tensile strain of the fiber, but is also not conducive to the collection of fibers.

The environmental parameters of electrospinning mainly include the temperature and humidity of the spinning environment. With the increase in temperature, the evaporation rate of the solvent in the spinning solution will also increase; generally, the viscosity of the spinning solution will also decrease with the increase in temperature, and it is also possible to obtain hollow nanofibers by increasing the temperature [88]. Humidity affects the evaporation rate of the solvent, and too high humidity will lead to incomplete drying of the solvent in the nanofibers.

## 3. Amino Acid

### 3.1. Introduction to Amino Acids

**Figure 6:**
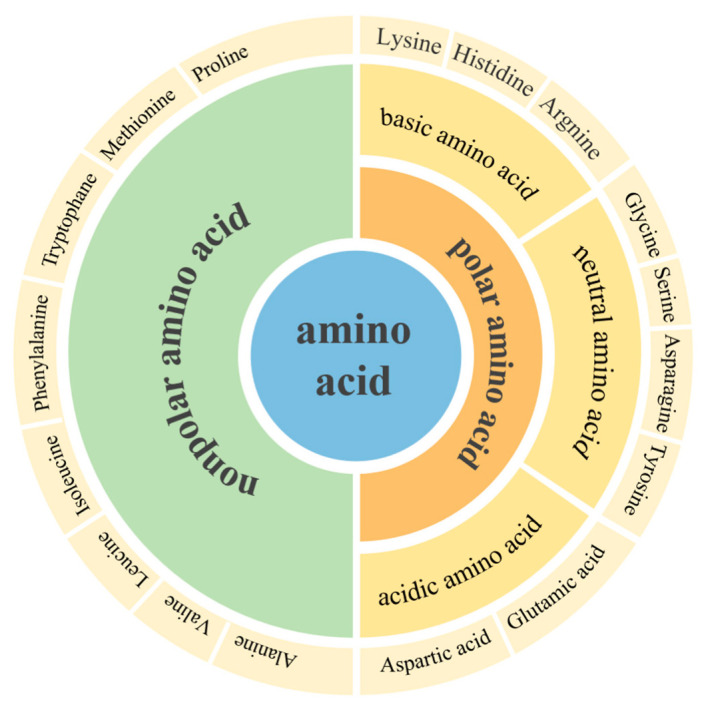
Amino acids and classification.

Amino acids are the basic unit of biological functional macromolecular proteins and the basic material of proteins required by animal nutrition. Amino acids are classified according to their side-chain groups and can be divided into polar amino acids and non-polar amino acids, of which polar amino acids can be divided into basic amino acids, acidic amino acids, and neutral amino acids [89], as shown in Figure 6. Poly amino acids are a kind of natural protein mimic, which have the same primary structure as natural proteins and polypeptide materials, and some poly amino acids can also simulate their secondary structure. With the diversity of amino acid structures, unique self-assembly structures and conformational transformation, high bioactivity, and good biocompatibility, they are widely used in the field of biomaterials. With the rapid increase in the application of polymer materials in the biomedical field, amino acid-derived functional polymers with the characteristics of degradability and high biocompatibility have also been vigorously developed. Because amino acids have bioactive side groups and diverse functional groups, they can realize a variety of functional polymerization, so they also have potential applications in imaging, drug delivery, cell adhesion, biodegradation, and so on [90,91]. 

### 3.2. Polar Amino Acids

#### 3.2.1. Basic Amino Acid

Basic amino acids are amino acids with hydroxide negative ions generated by hydrolysis with multiple hydrogen positive ions, including arginine, lysine, and histidine. The side chains of this class of amino acids contain protonatable basic groups

**Figure 7:**
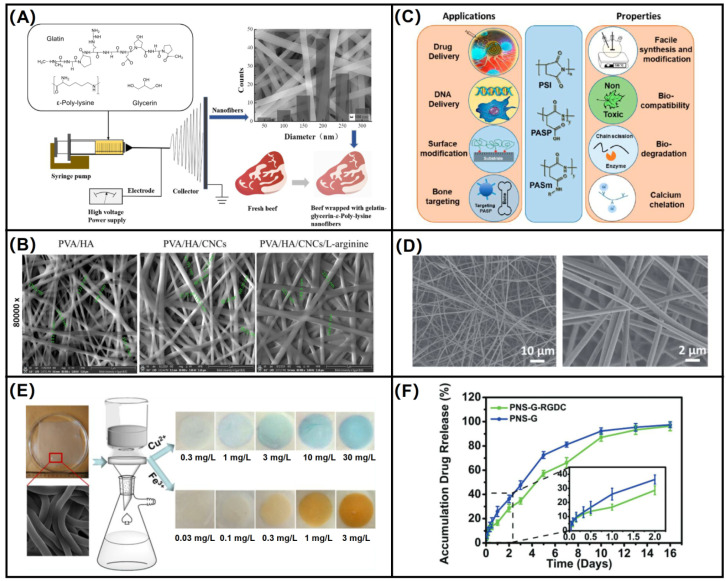
Preparation and application of polar amino acid electrospun nanofibers. (**A**) Electrospun gelatin-glycerin-ε-poly-lysine nanofibers [95]; (**B**) SEM images of selected PVA/HA, PVA/HA/CNCs, and PVA/HA/CNCs/L-arginine NFs scaffolds [98]; (**C**) properties and biomedical applications of poly(aspartic acid) and its derivatives [111]; (**D**) SEM image of electrospun nanofiber [114]; (**E**) application of procedure of PASP [112]; (**F**) Release curve of GS-Rg3 from fiber [106].

Lysine (Lys) is a common amino acid. Lys, which is water-soluble, biodegradable, and non-cytotoxic, is able to effectively promote cell adhesion and proliferation at the interface of biomaterials and improve tissue regeneration. In biological media, as the amine group on the Lys molecule is susceptible to protonation, it interacts with negatively charged cell membranes. Lys and its derivatives play an important role in biomedicine and tissue engineering [92,93,94]. Poly ε-lysine (ε-PL) can be produced by *Streptomyces albus* and has a variable number of L-lysine residues that are chemically bonded through an amide bond between the ε-amino and α-carboxyl groups. Broad-spectrum antibacterial activity is an important characteristic of poly-L-lysine, which can act as an antibacterial agent against bacteria, fungi, and even viruses. ε-PL was introduced into polyacrylic acid (PAA)/polyvinyl alcohol (PVA) electrospun nanofibers by Amariei et al. [95] for electrospinning together. Based on the antibacterial properties of ε-PL, the bacterial colonization rate of the ε-PL was reduced by several hundred times compared to unfunctionalized dressings, which later also showed good biocompatibility after cytotoxicity testing. Lin et al. [92] blended poly-L-lysine with gelatin and glycerol to make nanofiber mats by electrospinning, and the preparation process is shown in Figure 7A, where the addition of glycerol improved the mechanical strength of gelatin nanofibers and the addition of poly-L-lysine not only inhibited the growth of microorganisms but also extended the shelf life of food products, which is a promising food packaging material. Hyperbranched poly-L-lysine (HPLys) with low shrinkage can be used as a 3D scaffold because of its special properties, and it can be used in heart tissue engineering, promotion of cell adhesion, and good biocompatibility. As a scaffold material used in the heart, polyaniline nanofibers are widely used, but their biocompatibility is insufficient. Fernandes et al. [96] used HPLys to modify polyaniline and blended them together for electrospinning to prepare nanofibers. 

Arginine is often used as an additive to produce a facilitative effect on wound healing rather than a polymeric scaffold. Studies have shown that wound dressings loaded with arginine produce proline, which is required for collagen synthesis, promote the production of biologically active molecules in the body [97]. The biocompatibility and longevity of wound dressings can be enhanced by doping with arginine and controlling its sustained release. Hussein et al. [98] loaded arginine onto nanofibers made from a blend of PVA and hyaluronic acid to improve the uniformity of nanofiber diameter, and the SEM size and morphology of the prepared nanofibers are shown in Figure 7B. Exhibiting excellent hemocompatibility and outstanding proliferation and adhesion potential, especially against *Klebsiella pneumonia* showing sufficient antibacterial activity, this composite nanofiber could be further developed as a promising multifunctional wound dressing.

#### 3.2.2. Acidic Amino Acid

Poly(γ-glutamate) (γ-PGA) is an anionic poly(amino acid) and can be naturally produced by microorganisms such as *Bacillus subtilis*. γ-glutamyl transpeptidase in the human body can degrade γ-PGA to glutamate, which is non-toxic to the human body and widely present in the human body. γ-PGA has been gradually studied in biomedical fields, such as drug delivery, bioadhesion, and so on, because of its good biocompatibility, versatility, biodegradability, and high water retention [99,100,101]. However, the high solubility and fast dissolution rate of γ-PGA in water are not favorable for application in fields such as tissue repair scaffolds. So, in this case, cross-linking agents are needed to enhance the physical properties of γ-PGA for better application in the human body and biomedical field. Several common cross-linking agents such as ethylene glycol diglycidyl ether, cystamine [102], oxazoline (OXA) [103], and L-lysine [104] are able to cross-link γ-PGA and improve its stability in water. In addition, several studies have shown that electrostatically spun γ-PGA or γ-PGA-based composite fiber mats have good cell adhesion and proliferation ability. Lee et al. [105] innovatively co-blended PGA, polyethylene glycol (PEG), and TritonX-100 and were the first to successfully prepare PGA nanofibers by the electrospinning method. To overcome the water solubility of PGA, they chose to use butyl PGA for electrospinning and finally obtained water-insoluble nanofibers. Tajima et al. [103] chose OXA as the cross-linking agent; when the ratio of γ-PGA/OXA was 60/40% wt, the prepared nanofiber films could achieve skin-like tensile properties. Lower crosslinker blending ratios lead to higher hygroscopicity or water absorption, and due to the good mechanical properties and hygroscopicity of γ-PGA, the nanofibers prepared from it can be used in agrochemicals to biomedical products, which have a wide scope of application. Xu et al. [106] synthesized γ-PGA with L-cysteine and norbornene to synthesize photocrosslinkable γ-PGA-Nor. Due to the good mechanical properties and spinnability of PEO, it was introduced as a carrier polymer to facilitate the formation of electrospun fibers. The non-crosslinked fibers were prepared by electrospinning and finally crosslinked by UV irradiation to functionalize the fiber surface. This special nanofiber network has a structure and size similar to natural ECM, with ideal mechanical strength to support cell adhesion and promote growth on it, and has a significant inhibitory effect on proliferative scarring. The drug release curve of ginsenoside Rg3 (GS-Rg3) loaded on the fiber is shown in Figure 7F.

Poly(aspartic acid) (PASP) is synthesized by thermal polymerization of aspartic acid, a polyamino acid with carboxylic acid side chains, which is biodegradable, chelating, and dispersing. It has good solubility in most pH solutions, including the oral cavity with pH = 6.8, and is a degradable polymer with good water solubility [107]. It has been shown that poly(aspartic acid) is a polymer that can replace acrylic acid and is greener and more economical [108,109,110]. Due to its versatility such as water solubility and biodegradability, PASP has great applications in water treatment, wound dressing, stent material, drug delivery and release, etc. In addition, PSAP has functionalized carboxylic acid residues, which can be grafted with some polymers to improve the surface properties of the materials and can be applied to the fields of targeted regulation, antifouling, tissue engineering, and so on [111]. Figure 7C is a summary of some characteristics of PASP and its application areas. PASP nanofibers are generally prepared by choosing polysuccinimide (PSI), obtained from L-Asp catalyzed by phosphoric acid, as the precursor, and then cross-linking PSI nanofibers by hydrolysis to obtain PASP nanofibers. Zhang et al. [112] used the excellent adsorption of metal ions by PASP, combined with the high specific surface area of nanofibers, to design a sensor that can detect metal ions and can be reused, as shown in Figure 7E, and designed an electrospun nanofiber hydrogel membrane (ENHM) using PASP, which can be used as an aqueous solution colorimetric sensor for applications such as water treatment. Monlar et al. [113] electrospun PSI to obtain homogeneous nanofibers, which were then put into an imidazole-based buffer solution with pH = 8 for hydrolysis to obtain PASP gel fibers. This method produced PASP nanofibers similar to human ECM with desirable mechanical and biological properties. In 2017, Monlar et al. [114] again used coaxial electrospinning to improve the crosslinking process, using PSI as the shell polymer and adding PEO co-blending to improve the viscosity of the spinning solution and improve the stability of the jet. The crosslinking of 2,2,4 (2,4,4)-trimethyl-1,6-hexanediamine (THD) in the core layer of the nozzle occurs when the two solutions come into contact at the nozzle tip, forming PASP. This core-shell structure of nanofibers can effectively control the crosslinking time of the two solutions and complete the crosslinking with maximum efficiency, without clogging the nozzle, which is an effective method to prepare PASP nanofibers. The SEM image of nanofiber is shown in Figure 7D. This insoluble gel fiber is pH responsive and can be further applied in the future in the fields of drug release and tissue engineering.

#### 3.2.3. Neutral Amino Acid

Glycine is an important amino acid in the human body and is involved in the production of many important reactions and substances in the human body, including the production of DNA, proteins, and heme, as well as playing an important role in lipid metabolism, immune regulation, and neurotransmission. For this reason, glycine is often used to improve immunity and anti-inflammation, promote wound healing, and even improve neurological function, and has good biocompatibility, biodegradability, and excellent mechanical properties [115]. Alazzawi et al. [116] prepared nanofibers by electrospinning by blending PVA with an aqueous glycine solution. Such nanofibers with high specific surface area can be well used in applications such as bio-scaffolds and drug transport.

The electrospinning conditions and characteristics of several polar amino acids are shown in Table 2.

### 3.3. Nonpolar Amino Acids

**Figure 8:**
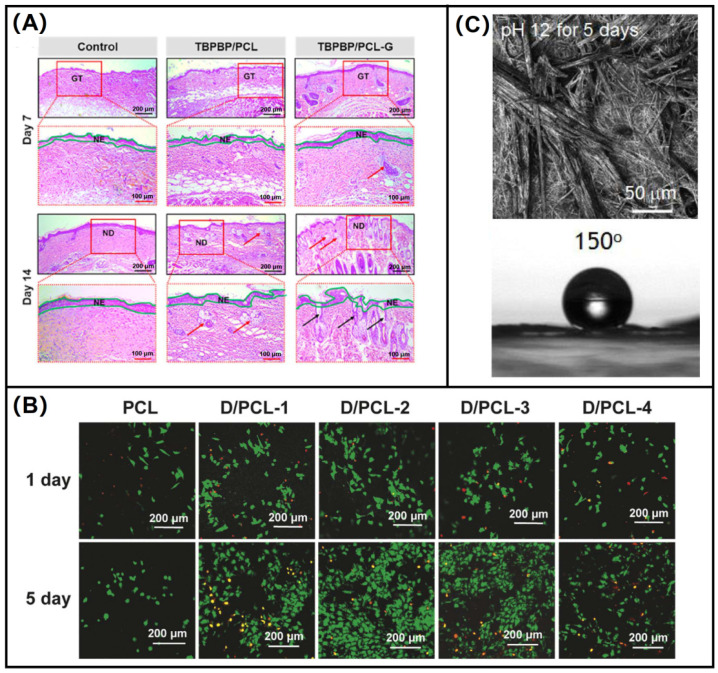
Application effect of nonpolar amino acid electrospun nanofibers. (**A**) H&E staining of the wound section, NE: new epidermis, GT: granulation tissue, ND: new dermis [123]; (**B**) confocal laser scanning microscope images of HUVECs cultured on chiral hybrid scaffolds [124]; (**C**) morphology and CA of PolyPhe nonwoven fabric after alkali treatment [125].

Phenylalanine is the most hydrophobic amino acid, and biodegradable superhydrophobic nonwoven materials can be obtained by electrospinning polyphenylalanine. Poly(L-phenylalanine) (PolyPhe) has a stable chemical structure and exhibits good chemical stability in both acidic and alkaline environments [121,122,123]. Sun et al. [124] electrospun chiral phenylalanine gels with PCL co-blended to prepare hybrid scaffolds, which can mimic human vascular endothelial cells, enhance cell adhesion, and promote vascular endothelial remodeling, which is an innovative approach for cardiovascular therapy. Confocal laser scanning microscopy images of HUVECs cultured on a chiral hybrid scaffold are shown in Figure 8A. The good biocompatibility of this material can be observed by staining live and dead cells green and red. Yoshida et al. [125] chose polyphenylalanine to prepare nanofibrous membranes by electrospinning. This electrospinning produced a nonwoven fabric that is superhydrophobic and biodegradable, which is promising for further wastewater treatment as well as biomedical applications. Chemical stability of PolyPhe nonwovens prepared by electrospinning into hexane/CHCl_3_ in basic (pH 12) conditions and the water contact angle (CA) of a water droplet on the PolyPhe nonwovens after the alkali treatment are shown in Figure 8B. In addition, chiral phenylalanine is often used as a gelling agent to enhance the cytocompatibility of polymers, and blending phenylalanine gels with polymers can modulate the biocompatibility of polymers.

Polyalanine acid, especially poly-b-alanine (PBA), contains amide bonds in its main chain, similar to protein molecules. Because PBA has a highly crystalline polymer structure, it is excellent in terms of heat resistance and mechanical properties, so it has excellent results in biomedical applications, is biologically active, and supports cell adhesion [126]. Catiker et al. [127] blended PBA with an optically active, biocompatible biodegradable thermoplastic poly(3-hydroxybutyrate) (P3HB). The addition of PBA enhances the opening of functional groups on the surface of P3HB and increases the biocompatibility and scaffolding properties of P3HB. The nanofibers prepared by the electrospinning method have a porous structure and can be applied to soft tissues.

Tryptophan is an essential amino acid that is involved in protein synthesis and is a precursor to many biologically active components in many important physiological activities. Tryptophan is also frequently used for medical diagnosis at the molecular level, including cataracts, colon tumors, renal cell carcinomas, etc. [128,129]. Li et al. [123] synthesized poly(esterurea)TP-PEU from tryptophan and L-phenylalanine, and then made nanofiber mats by electrospinning, loaded with S-nitrosoglutathione, which can release nitric oxide (NO) from S-nitrosoglutathione (GSNO). H&E staining of the wound section is shown in Figure 8C. The release of NO enables the treatment of wound infection and promotes wound cell proliferation and improves collagen deposition, which serves to promote wound healing.

The electrospinning conditions and characteristics of several nonpolar amino acids are shown in Table 3.

## 4. Application

### 4.1. Application in Hemostasis and Wound Healing

Wound injury caused by trauma or surgery is one of the most common clinical diseases, which has a great impact on the life of patients. Avoiding the serious consequences caused by excessive blood loss and wound infection has always been the focus of clinical medical research. It can be seen from the above that electrospinning nanofibers are a good choice with structural and performance advantages [130,131,132,133,134]. Electrospinning of poly(amino acids) with high biocompatibility and loading of drugs or active substances with anti-inflammatory, antibacterial, and hemostatic functions can obtain electrospun nanofibers with good medical effects and sustained drug release therapeutic effects [135,136,137,138,139].

**Figure 9:**
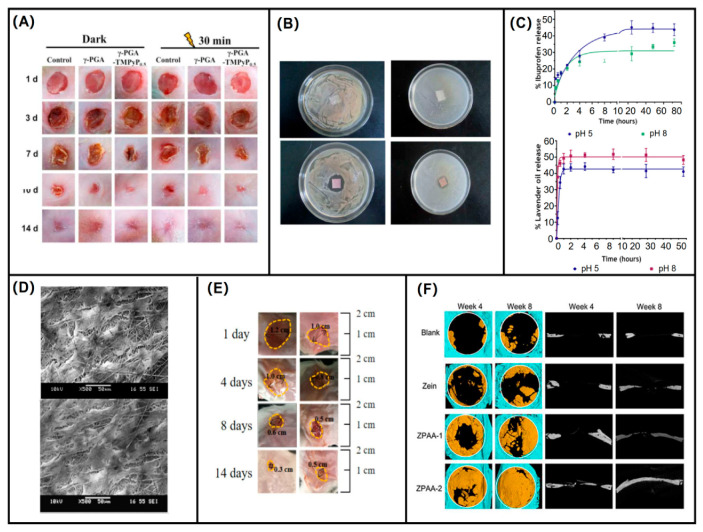
Application effect of poly(amino acid) electrospun nanofibers. (**A**) Photographs of wounds at different time intervals after treatment with different environments and materials [140]; (**B**) bacterial growth inhibition halos against *Staphylococcus aureus* and *E. coli* for PVA/L-H and PVA/L-H/Ag nanofibers [141]; (**C**) characterization of the IBP and LO in vitro release profile at pH 5 and pH 8 [142]; (**D**) SEM images showing the cell–biomaterial interactions on day 10 and day 15 on PLLA/PAA/Col I&III nanofibers [144]; (**E**) wound healing in the experimental and control groups at different times [149]; (**F**) three-dimensional micro-computed tomography reconstructed images of PAsp nanofibrous membrane treatment at 4 and 8 weeks postoperatively, the diameter of calvarial-defect model is 6 mm [159].

The nanofiber membranes obtained by electrospinning of amino acid polymers exhibit strong biocompatibility and have important applications in hemostasis and wound healing. Sun et al. [140] blended γ-PGA and cationic photosensitizer 5,10,15,20-tetrakis(1methylpyridinium-4-yl)porphyrin tetra (p-toluenesulfonate) (TMPyP) for electrospinning and stabilized them by chemical cross-linking. The treatment of wounds with this material is shown in Figure 9A. Cheng et al. [141] prepared ultrafine nanofibers of PVA/histidine/AgNPs for application in antimicrobial wound dressings. Since AgNPs are easily decomposed in polymer solutions, the researchers designed two separate tubes for PVA and histidine/AgNPs and mixed them only near the spinning head to further reduce the degradation rate of AgNPs and maximize their antimicrobial activity, and the antimicrobial effect of this wound dressing is shown in Figure 9B. This composite nanofiber is a convenient and efficient low-cost method, which is important for wound antimicrobial as well as further drug delivery. Sequeira et al. [142] prepared electrospun nanofibers by co-blending PVA and lysine, and also blended the anti-inflammatory agent ibuprofen (IBP) and antimicrobial agent lavender oil (LO) into the electrospun membrane by mixed electrospinning and surface physical adsorption methods. It was able to achieve sustained release of IBU and initial burst release of LO, as shown in Figure 9C, which is important for initial antimicrobial and sustained anti-inflammatory in the healing process of wounds. It is a novel composite wound dressing and also provides the possibility of further application of lysine and other amino acids. The prepared nanofiber mats can keep the wound environment moist and release cytotoxic reactive oxygen species (ROS) under visible light irradiation, which is amino acid material with bactericidal activity. It has good application potential for wound infection and wound healing. It has good potential for application in wound infection and wound healing. Nemeth et al. [143] performed solvent electrospinning of polyaspartic acid using ethanol as a solvent to design polyaspartic acid nanofibers capable of rapid dissolution in the oral pH environment and was shown to be a model with potential applications for sublingual drug delivery systems with potential applications in sublingual and oral drug delivery.

Ravichandran et al. [144] constructed a composite nanofiber scaffold composed of PLLA and polyaspartic acid, which not only mimics the natural ECM structure but also effectively mimics its biological function to enhance granulation tissue formation and guide new cells into the wound area, as shown in Figure 9D. This composite nanofiber membrane showed good biocompatibility on both days 10 and 15, which has a positive impact on promotion. Murase et al. [122] co-blended phenylalanine, adipic acid, and butylene glycol to make ester amide, and then made nanofibers by electrospinning method. This material is biocompatible and the resulting nanofiber mats degrade at a controlled rate. Due to the highly hydrophobic benzyl side group of phenylalanine, this ester amide is able to exhibit enhanced enzymatic hydrolysis ability. In addition, this composite fiber has an advantage in the field of drug delivery, as it was shown that this composite fiber can modulate its antimicrobial activity by drug loading according to the loading colchicine protease experiments.

### 4.2. Application in Special Trauma Repair

Burns are a major global public health problem, and burn trauma can be caused by heat (flame or scald), freezing, electricity, chemicals, radiation, or friction. Skin repair after burns requires a complex process following trauma, including coagulation, inflammation, cell proliferation, and tissue remodeling [145,146]. Burns can cause damage to the protective skin barrier and lead to pathogen colonization of the burn wound site. Burns can cause damage to body tissues and require polymers, bioactive molecules, or combinations thereof that support tissue regeneration [147,148]. The focus of the response to severe skin damage is to develop tissue skin substitutes that are biocompatible, sufficiently biodegradable, and hydrophilic, while electrospun nanofibers are good materials with an ECM-like structure, appropriate mechanical properties, and sufficient porosity. Fang et al. [149] used γ-PGA as the core solution and PLA as the shell solution to prepare coaxial nanofibers with a core-shell structure. Due to the good biocompatibility and biodegradability of PLA, γ-PGA is easy to process, easy to form film, and has good plasticity; it is readily biodegradable, promotes cell adhesion, and has slow-release properties. The animal model evaluation of the wound healing-promoting ability of γ-PGA/PLA fiber mat showed that this core-shell structured nanofiber has excellent wound healing-promoting ability and has great potential in the field of wound repair, as shown in Figure 9E.

Diabetes is a chronic degenerative disease. The diabetic foot is a symptom of diabetes, including infection, ulceration, or destruction of foot tissue, which seriously affects the quality of life of diabetics and, in more severe cases, requires amputation. Even if the diabetic foot heals, the chances of recurrence are very high. Diabetic wounds are often difficult to treat because they can become trapped in a vicious inflammatory cycle, where the response to growth factors is impaired, resulting in poor circulation and the continued release of inflammatory cytokines, which can produce excess amounts of reactive oxygen species, injuring microvasculature and creating other complications. Normal wounds can naturally terminate inflammation and heel, but diabetic wounds require appropriate treatment to enter a normal healing process [150]. Traditional diabetic foot dressings are primarily used to protect the wound from infection and do not aid in wound healing, and as technology advances active wound dressings that provide bioactive substances have been found to be more effective in treating diabetic foot wounds. Based on the similar structure of electrospinning and ECM, electrospinning of bioactive substances with biocompatible polymers is a feasible approach for the treatment of diabetic foot [151,152]. Isela et al. [153] used γ-PGA and polyvinyl alcohol (PVA) blended to prepare electrospinning nanofibers for wound dressing for diabetic foot treatment. The nature and fiber structure of γ-PGA and PVA provided a good base for the drug release system, which was able to sustain the release in phosphate-buffered medium for more than 200 h. This composite fiber is an effective model for the treatment of diabetic foot and also provides inspiration for the treatment of wounds in other sites.

Bone is actually an active tissue and can be reconfigured. Conventional scaffolds, made primarily of metal, calcium phosphate ceramic, or glass, have osteoconductive properties but not osteoinductive properties. They may also release toxic metal ions through corrosion or wear, leading to inflammation and allergic reactions. In addition, the non-biodegradable nature of such scaffolds in the natural environment further limits the repair of these challenging defects. An ideal bone regeneration scaffold should mimic ECM well, be biocompatible to promote cell growth, and have mechanical properties close to those of bone. Bone regeneration materials made of inorganic and metallic materials are less biocompatible and less flexible due to their high stiffness, in contrast to polymeric materials, which are more suitable. Polymer fibers can also be specially tailored to meet clinical needs. Composite polymer fibers prepared by electrospinning have been widely used for the treatment of bone, cartilage, and osteochondral defects because of their good bioactivity and superior mechanical properties. Bone regeneration membranes are commonly used in the treatment of bone tissue defects, and they are particularly suitable for recovery in cases of large bone injuries and massive bone grafts. Barrier membranes for bone regeneration applications play a key role in preventing cell entry from surrounding epithelial and connective tissues, preventing the proliferation of bone progenitor cells and formation of new bone tissue within the implant [154,155]. Common bone regeneration membranes are divided into two categories: resorbable membranes, which may cause damage to soft tissues and require secondary surgical removal, and non-resorbable membranes, which are generally chosen to use naturally degradable components that are friendly to patients and wound tissues but need to overcome the limitations of the mechanical properties of naturally degradable materials [156,157,158]. Liu et al. [159] used polyaspartic acid to modify maize proteins with good film-forming and biocompatibility properties. The nanofibrous membrane was made by electrospinning of maize protein, which has good film formation, biocompatibility, and mechanical properties. The nanofibrous membranes were implanted into intracranial bone defects in rats, and their ability to promote bone growth and repair was evaluated, as shown in Figure 9F. The zeatin nanofibrous membrane modified with polyaspartic acid has low cytotoxicity and provides a good microenvironment for promoting osteogenicity, which is a good biomaterial for coping with bone repair and has promising applications. Alazzawi et al. [116] prepared core-shell structured nanofibers using PCL as the shell and glycine and PVA as the core and wrapped glycine as the core part in them by coaxial electrospinning. This core-shell structured nanofiber is able to guide bone regeneration and promote the growth of bone defects, which is an ideal membrane for bone regeneration and can alleviate the rapid degradability of glycine. It is thus clear that composite materials of glycine and PVA have great potential for biomedical applications, and the choice of different additive materials may also have different effects on wound treatment. Poly(γ-glutamic acid)/β-tricalcium phosphate (γ-PGA/β-TCP) composite fiber mats were prepared by Yao et al. [160] using the electrospinning technique. Later, in the presence of 1-ethyl-(3-dimethylaminopropyl) carbodiimide (EDC), it reacted with cystamine to obtain a cross-linked product with improved water resistance, which can be better used as a new bone substitute. In addition, the cross-linked nanofibers tend to have higher alkaline phosphatase activity and are better able to promote cell adhesion, which are promising for applications not only in bone repair but also in tissue engineering.

## 5. Summary and Outlook

Nanofibers are widely researched and applied because of their nanoscale size which can well mimic the human ECM structure, as well as their porosity, high specific surface area, and other characteristics with good air permeability and moisture retention. Electrospinning, as an important method to prepare nanofibers, is also a relevant research hotspot in recent years. Single polymer electrospinning can no longer meet the requirements for hemostatic dressings nowadays, and a variety of polymers with compatible or complementary properties are selected to be blended with inorganic nanoparticles, drugs, and bioactive substances at the same time, and a variety of new electrospinning techniques are combined to design nanofibers with parallel, coaxial, and triaxial structures, which can be used in the fields of drug transport, drug controlled release, scar reduction, active substance encapsulation, etc. They have been applied in the fields of drug transport, drug release control, scar reduction, and active substance encapsulation, and can help wound healing in various aspects, such as hemostasis, antibacterial, and accelerated healing.

Amino acids, as a kind of biological small molecules, are often used in biomedical applications. Common amino acids such as glutamic acid, lysine, and aspartic acid are considered biomedical materials with the potential for development due to their excellent biocompatibility and biodegradability. At present, wound hemostatic dressings prepared by electrospinning of various amino acids are still relatively few and are mostly used to promote wound healing, and there are few amino acid composite fibers for wound hemostasis. From the above description and summary of amino acid wound dressings, it can be seen that the method of preparing electrospun nanofibers by blending amino acids with polymers is feasible, and if further addition of hemostatic materials, drugs, and bioactive substances may be able to achieve the desired hemostatic effect. The research on amino acid polymer materials in the field of electrospinning is not sufficient, and the electrospinning conditions of most amino acid polymers need to be further clarified. With the increasing attention and research on biomaterials in recent years, nanofibers based on amino acid polymers are gaining more and more attention, and further applications of amino acid polymers in electrospinning in various fields are also contemporary.

## Figures and Tables

**Table 1:** Key parameters affecting electrospinning.

Spinning solution properties	Polymer molecular weight	The fiber diameter increases with the increase in polymer molecular weight
	Solvent evaporation rate	The faster the solvent evaporates, the greater the fiber diameter; the faster the solvent evaporates, the greater the fiber porosity and specific surface area; the slower the solvent evaporates, the less easy to remove the solvent residue
	Spinning solution viscosity	The higher the viscosity of the spinning solution, the easier it is to block the spinneret; the lower the viscosity, the smaller the fiber diameter, but too low will produce electrospray
	Conductivity of spinning solution	The larger the dielectric constant, the smaller the fiber diameter; the smaller the dielectric constant, the easier it is to produce beads of fiber
Spinning parameters	Spinning voltage	The higher the spinning voltage, the smaller the fiber size; too much voltage will lead to unstable spinning; too little voltage fiber diameter will be coarse, or even produce droplets
	Liquid feeding speed	The larger the flow rate, the larger the fiber diameter, too large will produce droplets; low feed rate spinning process is easy to interrupt
	Collector	Influence the three-dimensional structure and arrangement of the product
	Distance between spinning head and collecting plate	Spacing is too small solvent cannot be fully evaporated; spacing is too large to affect the electric field strength, but also make the fiber is not easy to deposit and fly into the air
Environmental parameters	Spinning environment temperature	Increasing the temperature increases the rate of solvent volatilization, and hollow nanofibers can be obtained by increasing the temperature
	Spinning environment humidity	Elevated humidity reduces the rate of solvent evaporation, and nanocrystalline films can be obtained by increasing humidity

**Table 2:** Electrospinning conditions and characteristics of common polar amino acids.

Amino Acids	Additional Polymer	Solvent	Electrospun Technique	Characteristic	Ref.
Lysine	Gelatin/glycerin	Acetic acid	Blend	Excellent antibacterial ability against Listeria monocytogenes, a promising food packaging material	[92]
	PAA/PVA	Distilled water	Blend	Long-lasting antibacterial activity with good biocompatibility	[95]
	PAN	DCM/DMF	Blend	High biocompatibility and potential for culturing heart cells	[96]
	PAA	Distilled water	Blend	The addition of polylysine enhances the mechanical strength and stability of PAA	[117]
	PLLA/PPY	HFIP	Coaxial	Stable electrical properties, good biocompatibility, high cell adhesion rate	[118]
Glutamic acid	PLGA	TFA	Blend	Promotes wound healing and prevents tissue adhesions	[101]
	Cystamine (aftertreatment)	TFA	Blend	Good water stability	[102]
	OXA	Ethanol/water/ hydrochloric	Blend	Good mechanical properties and similar to skin, with certain moisture absorption properties	[103]
	PEG	Distilled water	Blend	Uniform nanofiber diameter	[105]
	PEO	Distilled water	Blend	Promotes cell adhesion and proliferation and inhibits proliferative scarring	[106]
	PVA	Distilled water	Blend	Promotes cell adhesion and can be used as a tissue engineering scaffold	[119]
	PCL	HFIP	Blend	Improves the solubility of florfenicol (FF) and promotes the in vitro release of the drug	[120]
Aspartic acid	PSI	—	Blend	Strong adsorption of metal ions and reduced water solubility after cross-linking, can be used as a colorimetric sensor for aqueous solutions	[112]
	PSI	DMF	Blend	A biocompatible fiber scaffold	[113]
	PSI/PEO/THD	DMF	Coaxial	pH sensitive for smart drug release applications	[114]
Arginine	PVA/HA	Distilled water	Blend	Accelerates wound healing and tissue regeneration	[98]
Glycine	PVA	Distilled water	Blend	High specific surface area for bio-scaffold and drug transport applications	[116]

**Table 3:** Electrospinning conditions and characteristics of common nonpolar amino acids.

Amino Acids	Additional Polymer	Solvent	Electrospun Technique	Characteristic	Ref.
Phenylalanine	PCL	HFIP	Blend	Cell adhesion is good and can be applied to vascular endothelial remodeling	[124]
	/	TFA/CHCl_3_	Blend	Super hydrophobic material to ensure stable adhesion of droplets	[125]
Alanine	P3HB	HFIP	Blend	Good biocompatibility and mechanical properties, conducive to cell adhesion and proliferation	[127]
Tryptophan	L-phenylalanine	HFIP	Blend	Treat wound infection and promote wound healing	[123]

## Data Availability

No applicable.
